# Ovarian cancer recurrence prediction: comparing confirmatory to real-world predictors with machine learning

**DOI:** 10.1016/j.esmorw.2025.100666

**Published:** 2026-01-08

**Authors:** D. Katsimpokis, A.E.C. van Odenhoven, M.A.J.M. van Erp, H.H.B. Wenzel, M.A. van der Aa, M.M.H. van Swieten, H.P.M. Smedts, J.M.J. Piek

**Affiliations:** 1 Department of Research & Development, Netherlands Comprehensive Cancer Organisation (IKNL), Utrecht; 2 GROW Research Institute for Oncology and Reproduction, Maastricht University, Maastricht; 3 Department of Gynaecology and Obstetrics and Catharina Cancer Institute, Catharina Hospital, Eindhoven; 4 Department of Gynaecology and Obstetrics, Amphia Hospital, Breda, The Netherlands

**Keywords:** ovarian cancer, cancer recurrence, prediction modeling, machine learning, real-world data

## Abstract

**Background:**

Ovarian cancer is one of the deadliest cancers in women, frequently diagnosed at an advanced stage, with a 5-year survival rate of 17%-28% in advanced-stage (International Federation of Gynecology and Obstetrics IIB-IV) disease. Machine learning (ML) may provide a better tool for survival prognosis than traditional methods and could provide insight into predictive factors. This study focuses on advanced-stage ovarian cancer and contrasts expert-derived predictive factors with data-driven ones from the Netherlands Cancer Registry (NCR) to predict progression-free survival.

**Materials and methods:**

A Delphi questionnaire was conducted to identify 14 predictive factors which were included in the final analysis. ML models (regularized Cox regression, random survival forests, and XGBoost) were used to compare the Delphi expert-based set of variables with a real-world data (RWD) variable set derived from the NCR. A non-regularized Cox model was used as the benchmark.

**Results:**

While regularized Cox models with the RWD variable set outperformed the traditional Cox regression with the Delphi variables (c-index: 0.70 versus 0.64, respectively), XGBoost showed the best performance overall (c-index: 0.75). The most predictive factors for recurrence, not identified by Delphi, were surgery type and debulking results, post-operative chemotherapy administration, number of platinum cycles, and socioeconomic status.

**Conclusions:**

Our results highlight that ML algorithms have higher predictive power compared with the traditional Cox regression. Moreover, RWD from a cancer registry identified more predictive variables than a panel of experts. Overall, these results have important implications for artificial intelligence (AI)-assisted clinical prognosis and provide insight into the differences between AI-driven and expert-based decision making in survival prediction.

## Highlights

• ML outperforms Cox regression in predicting ovarian cancer survival.
• Real-world predictors from the NCR outperform expert ones.
• XGBoost demonstrated the highest performance with a c-index of 0.75.
• Treatment variables and socioeconomic status are key predictors of recurrence.
• AI-driven methods show potential to enhance clinical prognostication.

## Introduction

Ovarian cancer is the fifth deadliest cancer in women worldwide and has the highest mortality rate of all gynecological cancers.^1^ In the Netherlands, ∼1300 women are diagnosed with ovarian cancer annually, of whom ∼1000 will die from the disease, which is usually diagnosed at an advanced stage.^2^ The 5-year survival rate is only 17%-28% for women with advanced-stage disease [International Federation of Gynecology and Obstetrics (FIGO) classification stages IIB-IV].^3^^,^^4^ Even after initial treatment, ∼70%-80% of patients develop recurrent disease.^5^

Despite decades of research on risk factors for ovarian cancer recurrence, such as FIGO stage and tumor characteristics, it is still challenging to accurately predict progression-free survival for (individual) ovarian cancer patients.^6^, ^7^, ^8^ In recent years, great promise has come from the field of artificial intelligence (AI) and machine learning (ML), where evidence for timely detection and accurate cancer prognosis has grown tremendously.^9^ By learning statistical associations from large amounts of data, ML models can show high accuracy of diagnostic prediction, sometimes comparable to or better than health care professionals (e.g. by spotting cancerous masses in magnetic resonance imaging data) and time-to-event modeling (e.g. overall/progression-free survival), thereby paving the way toward AI-assisted health care.^10^^,^^11^

Specifically in the field of gynecological cancers, the use of AI has also gained ground.^12^ For example, a recent meta-analysis of 34 imaging studies using AI to detect ovarian cancer across different imaging modalities showed that algorithms had equal and, sometimes, higher diagnostic accuracy than clinicians.^13^ ML models have also been developed to differentiate benign from malignant ovarian neoplasms, to predict overall survival after diagnosis, and to identify biomarkers and demographic factors contributing to overall survival.^14^, ^15^, ^16^ Most studies related to AI and ML in ovarian cancer have focused on the histopathology, diagnosis, and performance. However, prediction of progression-free survival and its related predictive factors has received less attention.^12^

The current study aims to identify predictive factors for ovarian cancer recurrence and evaluate the performance of ML models in comparison to traditional models, such as Cox regression. We specifically focused on patients with advanced-stage disease who face the most challenging survival profile. We featured two sets of predictive variables in our analysis: one set selected by medical experts through a Delphi consensus process and a larger set derived from real-world data (RWD) obtained from the Netherlands Cancer Registry (NCR). This work has important implications for AI-assisted clinical prognosis and provides insight into the differences between AI and medical expert-based variable selection for survival prediction.

## Materials and methods

### Data source

We extracted data on ovarian cancer from the NCR, which is the national population-based cancer registry of the Netherlands, with nationwide coverage since 1989. The registry offers cancer incidence information, along with clinical, sociodemographic, and (primary) treatment data. Recurrence data were collected by trained data managers ∼5 years after the initial diagnosis. Information pertinent to the management of gynecologic oncology was extracted from patient records, including radiology reports, pathology findings, and specialists’ consultation notes. Recurrence was defined as any documented relapse (i.e. recurrence or progression) of local, regional, or distant disease. Information on vital status and date of death is updated annually using an automated link with the national Personal Records Database (BRP), and is now up to date until 1 February 2024. From the NCR database, 65 variables related to ovarian cancer were available. However, in our expanded model (i.e. Delphi and exploratory variables), ∼30 variables were selected. This selection was based on two key considerations. Firstly, we carried out a pre-selection to identify variables relevant to the Delphi questionnaire, as some variables deemed irrelevant for clinical applicability. Secondly, we excluded variables that were contaminated by overlap with other variables, ensuring that only the most informative and relevant variables were included in this model.

According to the Dutch Central Committee on Research Involving Human Subjects, no ethical approval is needed for this study, as it is a retrospective study, which uses data from the NCR. The NCR data were de-identified before use in this study.

### Data sample

**Figure 1:**
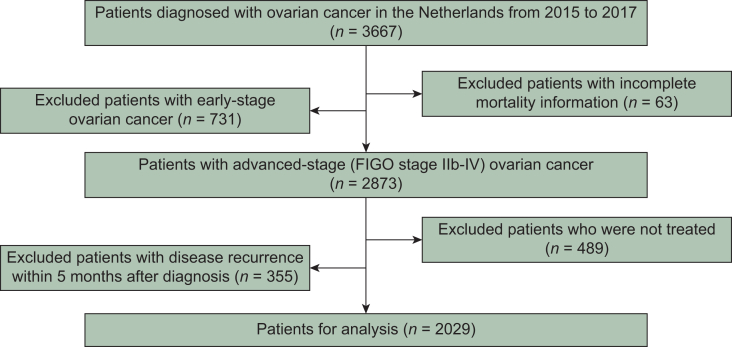
**Flow chart of patient selection.** FIGO, International Federation of Gynecology and Obstetrics.

Our initial sample consisted of patients diagnosed with epithelial ovarian cancer in the Netherlands from 2015 to 2017 (*n* = 3667). We excluded 63 patients with incomplete vital status information. We focused on advanced stages (FIGO stage IIB-IV; although some were clinically suspected of having early-stage disease), because these patients have a much higher mortality rate than those with early-stage disease, thereby further excluding 731 patients. Subsequently, we restricted the sample further in order to avoid two common biases. Firstly, we excluded 489 patients who did not receive any therapies, as it would act as a competing risk and, therefore, bias cumulative risk estimators.^17^ In addition, our sample included information on (the timing of) various types of therapy (i.e. neoadjuvant, primary, and adjuvant; primary and interval debulking surgery). Since some therapies can take longer to complete than others, they would present inflated survivorship estimates in comparison to the shorter therapy regimens only because people need to survive long enough to finish them, leading to immortal time bias.^18^ Our data showed evidence of immortal time bias (see Supplementary Appendix S2, available at https://doi.org/10.1016/j.esmorw.2025.100666). Since the vast majority (>85%) of the patients had completed their primary therapy within the first 5 months after diagnosis, we excluded an additional 355 patients who had disease recurrence before the first 5 months and we started following patients from 5 months after diagnosis. After exclusions, the final sample consisted of 2029 patients. A flow chart is provided in Figure 1.

### Delphi consensus making

A Delphi questionnaire was conducted to reach expert consensus.^19^ A total of 40 medical doctors (oncologists, gynecologic oncologists, surgical oncologists) were asked to participate, and 20 of them participated in the expert panel and filled out the questionnaires. We asked the participants to answer the following question: ‘In your experience as a medical specialist, what factors play a role in survival and disease-free survival in patients with high-stage (FIGO IIb-IV) ovarian cancer?’ Out of all responses, we identified 20 possible prognostic factors for recurrent disease. Information for six factors was not available in the NCR. Thus, 14 expert-identified prognostic factors were included in the analysis (see Table 1). Detailed information about the full Delphi process and the outcomes of the questionnaire are presented in the Supplementary Appendix S1, available at https://doi.org/10.1016/j.esmorw.2025.100666. All data were evaluated and analyzed by two clinical investigators (A.E.C.v.O., M.A.J.M.v.E.).

**Table 1:** Delphi variables agreed by experts as predictive factors, presented in alphabetical order

Delphi-suggested variables	Presence in the NCR	NCR-related variables
Age of the patient	✔	Age at diagnosis
Ascites	✔	—
CA 125 after chemotherapy	—	—
Chemotherapy–surgery in-between time	✔	Chemotherapy–surgery in-between time
Comorbidities	✔	CCI
Condition of the patient	✔	Performance status, ASA, CCI
Dosage chemotherapy complete	✔	Number of platinum cycles
FIGO classification	✔	FIGO classification
Genetic predisposition	✔	BRCA molecular diagnostics
Histological subtype of the tumor	✔	Histological subtype
Involvement of mesentery	—	—
Level of PCI	—	—
Motivation of the patient	—	—
Nutritional status	✔	BMI
Pattern of metastases (lymph nodes involved)	✔	Number of positive lymph nodes
Quality of surgical team	—	—
Response on chemotherapy	—	—
Time to recurrence	✔	Time to recurrence
Tumor biology	✔	*BRCA* molecular diagnostics
WHO performance status	✔	Performance status

The second column indicates the presence of information in the NCR. The last column lists NCR variables that encodes this information. ‘✔’ shows presence in the NCR, while ‘—’ shows absence or practically absence (>90%) of information in the NCR.

ASA, American Society of Anesthesiologists; BMI, body mass index; CA 125, cancer antigen 125; CCI, Charlson Comorbidity Index score; PCI, Peritoneal Cancer Index.

### Data variables

#### Delphi approach

This set includes 14 predictor variables from the Delphi consensus. These variables constitute the theoretical (i.e. expert-based) minimum number of predictors for progression-free ovarian cancer survival. These variables were defined without seeing the data, thereby constituting a confirmatory test.^20^

#### Data-driven approach

For the data-driven approach, we relied on a larger RWD set of prognostic factors. In this case, the following NCR variables were included on top of the Delphi variables: pretreatment CA 125, ovariectomy (no versus yes), ovariectomy with hysterectomy (no versus yes), staging surgery (no versus yes), result of debulking (complete versus optimal versus suboptimal versus no debulking), type of debulking (interval versus primary versus no debulking), type of chemotherapy (preoperative versus post-operative versus pre- and post-operative versus no chemotherapy versus yes, but no surgery), hormonal therapy (no versus yes), intraperitoneal chemotherapy (no versus yes), hyperthermic intraperitoneal chemotherapy (no versus yes), differentiation grade (levels 1-3), laterality (bilateral versus unilateral versus extra-ovarian), socioeconomic status (levels 1-10).

### Machine learning modeling

#### Cox-based models

For the Delphi approach, we used the semi-parametric Cox proportional hazards model with the Delphi variables. This makes the model easy to interpret because it remains agnostic about the baseline hazard function while treating the predictors in a regression-type form. However, the model relies on proportional hazards assumptions, which can be restrictive and may not always hold true in practice.^21^

In our RWD approach, we applied two adaptations of the Cox model with the full set of variables: (i) the Cox model with the Lasso regularization and (ii) the Cox model with the Elastic Net, which combines Lasso and Ridge regularizations. Regularization is crucial for two reasons. Firstly, it has been shown to enhance the performance of the Cox model.^22^^,^^23^ Secondly, it prevents overfitting by shrinking non-predictive coefficients to zero, effectively carrying out variable selection. However, compared with Lasso, Elastic Net tends to carry out more stable variable selection in the presence of correlated variables.^24^ Therefore, we included both models in subsequent analyses for comparison.

For this study, the development, validation, and reporting of the predictive model were conducted in accordance with the TRIPOD (Transparent Reporting of a multivariable prediction model for Individual Prognosis or Diagnosis) guidelines.^25^

#### Model fitting

The full model fitting procedure is described in Supplementary Appendix S2, available at https://doi.org/10.1016/j.esmorw.2025.100666. In short, missing values were imputed using the MissForest algorithm.^26^ The models were fitted using internal validation based on bootstrapping (*n* = 500) to adjust for generalization error.^27^ Model parameters were optimized during the internal validation step using a randomized cross-validation parameter search. Bootstrapping was used to achieve stable variable selection estimates.

#### More advanced machine learning modeling

While Cox-based models are easily interpretable, we also explored random survival forests (RSFs) and the extreme gradient boosting trees model (XGBoost), which have demonstrated state-of-the-art performance in order to evaluate their marginal predictive advantage within the RWD approach.^11^^,^^28^, ^29^, ^30^ Both RSFs and XGBoost are not restricted by the proportional hazards assumption, allowing them to capture non-linear relationships in the data. Moreover, since XGBoost can natively handle missing values, we fitted imputed and non-imputed versions of the RWD to this model to investigate the impact of imputation on performance. Supplementary Appendix S2, available at https://doi.org/10.1016/j.esmorw.2025.100666, provides more details.

## Results

Table 2 presents the descriptive statistics of the cohort (i.e. variables of the Delphi and the data-driven approach). Seventy-seven percent of patients (i.e. no recurrence = 467; recurrence = 1562) showed disease recurrence after the start of follow-up, which began 5 months after the initial diagnosis, with a mean time to recurrence of 600 days (standard deviation: 570 days) and a maximum follow-up time of 2043 days.

**Table 2:** Descriptive statistics of predictor variables (in alphabetical order)

Age of the patient (in years), mean (SD^a^)	66 (10.8)
ASA classification, *n* (%)	
I	295 (14)
II	1081 (53)
III	306 (15)
NA^a^	347 (17)
BMI^a^ (kg/m^2^), mean (SD)	25.8 (4.76)
*BRCA*^a^ mutation, *n* (%)	
*BRCA1*	106 (5)
*BRCA2*	66 (3)
No *BRCA* mutation	1056 (53)
No *BRCA* mutation analysis	336 (17)
NA	465 (23)
CCI,^a^*n* (%)	
0	1246 (61)
1	467 (23)
2 (two or more)	149 (7)
NA	167 (8)
Differentiation grade, *n* (%)	
1	141 (7)
2	86 (4)
3	1352 (67)
NA	450 (22)
Chemotherapy, *n* (%)	
No	76 (4)
Only post-operative	584 (29)
Pre- and post-operative	1019 (50)
Only preoperative	39 (19)
Yes, but no surgery	311 (15)
Time between chemotherapy and surgery (days), mean (SD)	42.7 (44.8)
Debulking result, *n* (%)	
Complete	1046 (52)
Optimal	428 (21)
Incomplete	121 (6)
No debulking	416 (21)
NA	18 (0.8)
Debulking type, *n* (%)	
Primary	565 (28)
Interval	1048 (52)
No debulking	416 (21)
NA	18 (0.8)
FIGO^a^ classification, *n* (%)	
IIB	209 (10)
III	1254 (62)
IV	566 (28)
WHO^a^ performance status, *n* (%)	
0	893 (43)
1	459 (23)
2	108 (5)
3	27 (1)
NA	542 (27)
Histological subtype of the tumor, *n* (%)	
Adenocarcinoma NOS^a^	173 (8)
Clear cell	83 (4)
Endometrioid	53 (3)
Mucinous	41 (2)
Other	74 (4)
Serous	1605 (79)
Hormonal therapy, *n* (%)	
No	1966 (97)
Yes	63 (3)
HIPEC,^a^*n* (%)	
No	2010 (99)
Yes	19 (1)
Ovariectomy with hysterectomy, *n* (%)	
No	2010 (99)
Yes	19 (1)
Intraperitoneal chemotherapy, *n* (%)	
No	1964 (97)
Yes	65 (3)
Staging surgery, *n* (%)	
No	1978 (97)
Yes	51 (3)
Laterality, *n* (%)	
Bilateral	827 (40)
Other	201 (10)
Unilateral	747 (39)
NA	254 (12)
Number of platinum based chemotherapy cycles, *n* (%)	
6	1522 (75)
<6	288 (15)
>6	219 (11)
Ovariectomy, *n* (%)	
No	1962 (97)
Yes	67 (3)
Pretreatment CA 125, mean (SD)	1613.7 (31113.1)
SES^a^ (deciles), *n* (%)	
1	217 (11)
2	211 (10)
3	234 (12)
4	221 (11)
5	227 (11)
6	200 (10)
7	191 (9)
8	182 (9)
9	173 (9)
10	164 (8)
NA	9 (<1)

In case of categorical or ordinal variables, the count and percentage of each level are given. In case of numerical variables the mean and SD are given.

ASA, American Society of Anesthesiologists; BMI, body mass index; *BRCA*, BReast CAncer gene; CA 125, cancer antigen-125; CCI, Charlson Comorbidity Index; FIGO, International Federation of Gynecology and Obstetrics; HIPEC, hyperthermic intraperitoneal chemotherapy; NA, not available; NOS, not otherwise specified; SD, standard deviation; SES, socioeconomic status; WHO, World Health Organization.

a Additional variables of the real-world data approach.

The comparison of the three Cox models indicated that the baseline (Delphi) model revealed lower predictive accuracy than the RWD approach (see Table 3). This shows that the additional cancer registry variables have an added predictive value beyond the Delphi variables. Conversely, some predictors identified in the Delphi process may not be very predictive in the presence of the RWD variable set. Although both regularization models achieved the same performance, we selected the Elastic Net model for variable selection, because it produces more stable coefficients than the Lasso model in the presence of correlated variables. Notably, calibration of the Elastic Net model indicated no gross misfit (see Supplementary Appendix S3, available at https://doi.org/10.1016/j.esmorw.2025.100666).

**Table 3:** Model performance adjusted for generalization error (i.e. overfitting) based on bootstrapping (500 iterations)

Model	Mean performance	95% CI
Cox model (baseline)	0.643	0.657-0.627
Cox model (Lasso)	0.703	0.689-0.717
Cox model (Elastic Net)	0.703	0.689-0.716
**XGBoost (without NAs)**	0.754	0.738-0.773
XGBoost (with NAs)	0.739	0.723-0.760
Random forests	0.721	0.708-0.735

Mean and 95% quantile-based confidence intervals (CIs) are given. Numbers indicate the concordance index (c-index). ‘NAs’ indicate whether the dataset contained missing values or whether these were imputed beforehand. Note that only the Cox model (baseline) was fitted to the Delphi variables alone, while the rest of the models were fitted to the real-world data variable set. The best-performing model is indicated in bold.

In comparison to the data-driven Cox-based models, the XGBoost model, which was also fitted on the RWD, achieved higher performance than the Lasso and Elastic Net models, both in the version with imputed data and in the version without data imputation (see Table 3). Data imputation did not change the performance of XGBoost, implying that the MissForest imputation method did not decrease performance. It is noteworthy that the XGBoost model showed a substantial improvement of performance over the baseline Cox regression model with the Delphi variables. Finally, RSFs scored lower than the XGBoost model, but performed better in comparison to the (regularized) Cox regression models.

**Figure 2:**
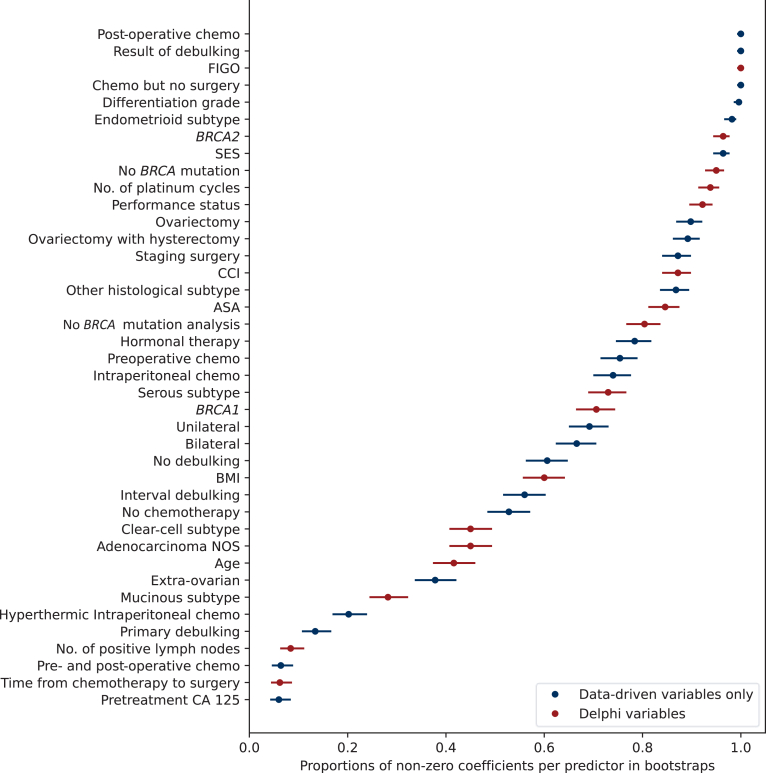
**Variable selection from the Elastic Net model.** Each dot indicates the proportion that each predictor was non-zero in the bootstrap samples, and the range indicates the estimated 95% Wilson-based confidence interval. The higher the percentage, the more important the variable is for prediction, and vice versa. ASA, American Society of Anesthesiologists; Chemo, chemotherapy; *BRCA*, BReast CAncer gene; CCI, Charlson Comorbidity Index; FIGO, International Federation of Gynecology and Obstetrics; NOS, not otherwise specified.

Variable selection showed a variety of results (see Figure 2). The top 10 predictive variables showed a mix of variables grouped into three categories, relating to (i) intervention type and outcome (i.e. type of surgery and results of debulking, presence of post-operative chemotherapy, number of platinum cycles), (ii) clinical characteristics (i.e. differentiation grade, FIGO stage, endometrioid cancer type, *BRCA* mutation), and (iii) demographics (i.e. socioeconomic status). Interestingly, only 5 of the top 10 variables were identified by the Delphi panel, namely FIGO stage, endometrioid cancer type, *BRCA2* or no *BRCA* mutation, and number of platinum cycles, highlighting the importance of the data-driven variables. Moreover, two of the Delphi variables, e.g. the number of positive lymph nodes and time from chemotherapy to surgery, had low predictive power regarding disease recurrence. Lastly, other variables identified by the Delphi consensus, such as age and body mass index, were found to be moderately predictive.

## Discussion

By directly comparing expert opinions (by the Delphi consensus) with a broader set of RWD, we show that data-driven variable selection captures crucial ovarian cancer recurrence predictors—especially treatment characteristics and socioeconomic factors—not identified by clinical experts. We applied and compared several ML models in order to predict progression-free survival of patients with ovarian cancer. We compared a confirmatory Delphi-based set of predictor variables with RWD drawn from the NCR that included the Delphi variables (i.e. nested comparison). Our results show that regularized Cox regression models based on the RWD variable set outperformed the traditional Cox regression based on the Delphi set (c-indices: 0.64 versus 0.70, respectively). This means that the traditional Cox regression would correctly discriminate the time-to-event of a random pair of patients only 64% of the time, in contrast to 70% for the regularized version based on the RWD set. Furthermore, variable selection on the regularized Cox model (i.e. Elastic Net) showed that many predictive variables for recurrence were related to types and results of therapy as well as socioeconomic status, all of which were not included in the Delphi set. Specifically, out of the 10 most predictive variables, only 5 variables were identified by the Delphi consensus. Thirdly, the state-of-the-art XGBoost model achieved the best performance (c-index: 0.75) on the RWD variable list, outperforming both regularized Cox regression models and RSFs.

The superior model performance of the RWD approach underlines the importance of using data-driven, cancer registry variables for predicting ovarian cancer recurrence. Although the Delphi variables represent the (minimal) expert-based set of expected predictive factors for recurrence, not all of them were highly predictive (e.g. age), and some showed little to no predictive value (e.g. number of cancer-positive lymph nodes). Furthermore, many factors not mentioned in the Delphi consensus showed a high degree of predictiveness (e.g. socioeconomic status). On the other hand, some variables that were not included in the Delphi consensus (e.g. pretreatment CA 125) played indeed a minimal role in recurrence prediction.

Our results indicate that while expert-based knowledge plays a valuable role in predicting ovarian cancer recurrence, it has limitations and should therefore be complemented with RWD from cancer registries. Future research can further enrich these datasets by incorporating primary health care data as well as more intricate demographic and time-varying information, to improve predictive accuracy. However, it is important to note that the predictive value of the factors analyzed in this study reflects the statistical correlations among these variables, rather than causal relationships. Establishing causality would require counterfactual intervention and a deeper understanding of the causal structure of the study system.^31^ Such causal understanding, which is absent from ML models, may further explain why variable selection results from ML models and from the Delphi questionnaire did not completely overlap. This research question, which is beyond the scope of the current paper, could shed light on the medical decision making during the Delphi process.

Variable selection through the Cox-based Elastic Net is easy to interpret. Due to their complexity, more advanced models such as RSFs and XGBoost require explainability methods to understand how the models use the predictor variables to carry out prediction. Techniques such as Shapley values^32^ can provide valuable insights into the contribution of each variable to the model’s prediction, but still require complex computations and are prone to overinterpretation (e.g. as causal indicators) or misinterpretation due to confirmation bias.^33^^,^^34^ In our current study, we opted to carry out variable selection with the easily explainable Cox-based Elastic Net, since the performance of the XGBoost model was only marginally higher than that of the Cox-based models.

The superior performance of the XGBoost model is in line with recent literature suggesting that the model can outperform Cox-based models and other ensemble methods, such as RSFs, for time-to-event modeling.^11^ Although the XGBoost model technically outperformed the regularized Cox regression models, its advantage in predictive performance was not substantial. This result may stem from the fact that the proportional hazards assumption of the Cox model seems to not be grossly violated in our data (e.g. the calibration of the Cox models was good; see Supplementary Figure S1, available at https://doi.org/10.1016/j.esmorw.2025.100666). Larger sample sizes (i.e. many tens of thousands of patient records) might be required for such non-linear patterns to be effectively used by XGBoost.

The current study has many methodological strengths. Firstly, the internal validation method based on bootstrapping validates the model building procedure and provides more stable performance metrics, including overfitting and variable selection, compared with simpler train–test split techniques.^27^ Secondly, our sample size is larger than that in previous studies and we used a wide range of predictive factors, ranging from demographic and clinical to primary therapy information, thereby employing the strength of NCR information.^35^^,^^36^ Thirdly, we used an a priori expert-informed set of variables (i.e. the Delphi consensus) in order to have a theoretical baseline for the minimal set of predictive factors for disease recurrence before we examine the data. This allowed us to separate confirmatory testing of this minimal set of variables from the expanded RWD set.

The study has also limitations. First of all, our model is only valid for prediction with follow-up starting at 5 months after diagnosis and for those patients who received at least some primary therapy, in order to avoid competing risk bias and immortal time bias, as the ML models used cannot natively correct for these. Future research could use more advanced methods, such as simulation to model competing risks. In addition, models such as time-varying-coefficient Cox regression could account for the different durations of the different combinations of primary therapies.^37^

A second limitation of the current study is that the variables identified by the Delphi consensus are not always available in the NCR (i.e. motivation of the patient, response on chemotherapy, level of Peritoneal Cancer Index, CA 125 levels after chemotherapy, involvement of mesentery and quality of surgical team, ascites). For example, motivation of the patient or quality of surgical team cannot be quantified easily, making it difficult to include. Patient-reported outcomes could serve as a proxy for quality of life and hospital experience, which would be an interesting avenue to explore in the future. In addition, in our current Delphi implementation, we did not inform experts about which and whether data variables are available in the NCR, because our intention was to elicit the ‘ground truth’ regarding progression-free survival unconditionally from data availability. Future studies could experiment with the Delphi design where experts could adjust their verdict at the final stage after having been informed about data availability or data missingness (or even additional data information) from the study data source. This could allow higher alignment of the RWD approach with the Delphi’s final verdict.

Thirdly, and perhaps more importantly, even our best-performing model achieved moderate performance for predicting ovarian cancer recurrence (with c-index at 0.754), making it difficult for usage in actual clinical practice. More complex models, such as deep neural networks, could be explored for better performance, although this would bring forth other challenges such as sample size limitations or explainability difficulties. Further evidence-based model comparisons can shed light on this topic.

### Conclusion

Our work provides evidence that ML algorithms, such as XGBoost, RSFs, and regularized Cox regression, are better in predicting progression-free survival for ovarian cancer than the traditional Cox model. In addition, expert-derived predictors can be enhanced through secondary (cancer registry) data, which can provide better predictive results when combined with ML variable selection. In doing so, the study offers a step forward in refining individualized recurrence risk estimation, which can help clinicians to stratify recurrence risk, adjust follow-up strategies, and inform shared decision making.
