# Supplementary material for: Ovarian cancer recurrence prediction: comparing confirmatory to real-world predictors with machine learning
Source: ESMO Real World Data Digit Oncol. 2026 Jan 8;11:100666. doi: 10.1016/j.esmorw.2025.100666 (PMC13040900; doi:10.1016/j.esmorw.2025.100666)
Supplement: Supplementary Material [file mmc1.docx]

**Supplementary**

**Delphi questionnaire**

**General information Delphi questionnaire p.2**

**Supplementary document 1.** *Questionnaires round 1 p.3*

**Supplementary document 2.** *Total feedback of all respondents of round 2 (main question one and two) p.5-6*

**Supplementary document 3.** *Total feedback of all respondents of round 3 (main question one and two) p.7-8*

**Supplementary document 4.** *Consensus statements p.9-10*

**General information Delphi questionnaire**

A Delphi process was employed to establish a list of prognostic factors for ovarian cancer recurrence. This method involved a panel of experts in ovarian cancer treatment and research.

First we selected a panel of medical specialists with experience in ovarian cancer. This selection included all medical specialists’ who work in the region of North Brabant. We have sent the first questionnaire (see supplementary 1.) to 40 possible participants.

The Delphi process involved three rounds in which experts individually and independently proposed prognostic factors and these were reviewed by the whole expert panel after each proposal round. Consensus was reached if the median score given to proposed prognostic factor by the panel was four or higher (supplementary 2 and 3). Eventually, twenty respondents completed all three rounds.

**Supplementary document 1**. Questionnaire round one Delphi

Questionnaire 1. Characteristics

- 1. Informed consent

1. Yes
2. No
   1. In what kind of center do you practice?
3. Academic center
4. Non-academic center
5. Research center
6. Tertiary center
   - 1. If you work in a non-academic center, please note whether there is surgery performed in your center?
7. We perform debulking surgery
8. We do not perform debulking surgery

1.3. How many years of experience do you have as a medical specialist?

1.4 On average, how many cases of high stage (FIGO IIb or higher) ovarian cancer do you see per year

1.5 In what ways do you work with patients with ovarian cancer? (multiple answers possible)

1. Head practitioner
2. Co-treater
3. Scientific research
4. Surgeon
5. I refer them to a specialized center for further treatment

Questionnaire 2. Two main questions with open answer:

1. In your experience as a medical specialists, what factors play a role in survival and disease free survival in ovarian carcinoma **FIGO-stage IIb or higher?**
2. In your experience as a medical specialists, what factors play a role in survival and disease free survival in ovarian carcinoma **regardless of the FIGO-stage?**

**Supplementary document 2a.** *Total feedback of all respondents of round two (main question one)*

In your experience as a medical specialists, what factors play a role in survival and disease free survival in ovarian carcinoma **FIGO-stage IIb or higher?**

|  | Disagree | | Neutral | Agree | | Votes | Median | Consensus |
| --- | --- | --- | --- | --- | --- | --- | --- | --- |
| Nr. Statement | 1 | 2 | 3 | 4 | 5 |  |  |  |
| 1 Age of the patient | 0 | 1 | 6 | 10 | 3 | 20 | 3,75 | No |
| 2 Condition of the patient | 0 | 0 | 1 | 6 | 13 | 20 | 4,6 | Agree |
| 3 Motivation of the patient | 0 | 3 | 4 | 7 | 6 | 20 | 3,8 | No |
| 4 Nutritional status | 0 | 0 | 0 | 16 | 4 | 20 | 4,2 | Agree |
| 5 Comorbidities | 0 | 0 | 0 | 13 | 7 | 20 | 4,35 | Agree |
| 6 WHO-performance status | 0 | 0 | 0 | 11 | 9 | 20 | 4,45 | Agree |
| 7 Genetic predisposition | 0 | 2 | 4 | 12 | 2 | 20 | 3,7 | No |
| 8 FIGO-classification | 0 | 0 | 1 | 9 | 10 | 20 | 3,45 | No |
| 9 Histological subtype of the tumor | 0 | 0 | 2 | 10 | 8 | 20 | 4,3 | Agree |
| 10 Tumor biology | 0 | 0 | 2 | 12 | 6 | 20 | 4,2 | Agree |
| 11 Time between chemotherapy and surgery | 1 | 3 | 4 | 8 | 4 | 20 | 3,55 | No |
| 12 Response on chemotherapy | 0 | 0 | 0 | 9 | 11 | 20 | 4,55 | Agree |
| 13 Dosage of chemotherapy complete | 0 | 4 | 4 | 11 | 1 | 20 | 3,45 | No |
| 14 Level of PCI | 0 | 2 | 2 | 8 | 8 | 20 | 4,1 | Agree |
| 15 CA-125 decrease after neoadjuvant chemotherapy | 0 | 1 | 1 | 13 | 5 | 20 | 4,1 | Agree |
| 16 CA-125 at diagnosis | 1 | 5 | 10 | 1 | 3 | 20 | 3 | No |
| 17 Ascites | 0 | 4 | 6 | 6 | 4 | 20 | 3,5 | No |
| 18 Involvement of mesentery | 0 | 2 | 6 | 3 | 9 | 20 | 3,95 | No |
| 19 Pattern of metastases (lymph nodes involved) | 0 | 1 | 2 | 13 | 4 | 20 | 4 | Agree |
| 20 Time to recurrence | 0 | 0 | 0 | 10 | 10 | 20 | 4,5 | Agree |
| 21 Quality of surgical team | 0 | 0 | 6 | 11 | 3 | 20 | 3,85 | No |

**Supplementary document 2b.** *Total feedback of all respondents of round two (main question two)*

In your experience as a medical specialists, what factors play a role in survival and disease free survival in ovarian carcinoma **regardless of the FIGO-stage**?

|  | Disagree | | Neutral | Agree | | Votes | Median | Consensus |
| --- | --- | --- | --- | --- | --- | --- | --- | --- |
| Nr. Statement | 1 | 2 | 3 | 4 | 5 |  |  |  |
| 1 Age of the patient | 0 | 1 | 3 | 10 | 6 | 20 | 4,05 | Agree |
| 2 Condition of the patient | 0 | 0 | 1 | 12 | 7 | 20 | 4,3 | Agree |
| 3 WHO-performance | 0 | 0 | 1 | 13 | 6 | 20 | 4,25 | Agree |
| 4 Comorbidities | 0 | 0 | 2 | 13 | 5 | 20 | 4,15 | Agree |
| 5 Smoking yes or no | 0 | 4 | 7 | 8 | 1 | 20 | 3,3 | No |
| 6 FIGO-classification | 0 | 0 | 0 | 9 | 11 | 20 | 4,55 | Agree |
| 7 Histological subtype of the tumor | 0 | 0 | 1 | 11 | 8 | 20 | 4,35 | Agree |
| 8 Tumor biology | 0 | 0 | 2 | 12 | 5 | 20 | 4,2 | Agree |
| 9 Genetic predisposition | 0 | 2 | 4 | 10 | 4 | 20 | 3,8 | No |
| 10 Primary debulking achievable | 0 | 0 | 6 | 7 | 7 | 20 | 4,05 | Agree |
| 11 Response on chemotherapy | 0 | 0 | 1 | 10 | 9 | 20 | 4,4 | Agree |
| 12 HIPEC | 0 | 1 | 10 | 7 | 2 | 20 | 3,5 | No |
| 13 Level of CA-125 at diagnosis | 1 | 3 | 7 | 7 | 2 | 20 | 3,3 | No |
| 14 CA-125 decrease after neoadjuvant chemotherapy | 0 | 0 | 2 | 15 | 3 | 20 | 4,05 | Agree |
| 15 Quality of surgical team | 0 | 0 | 5 | 12 | 3 | 20 | 3,9 | No |
| 16 Complete staging | 0 | 0 | 4 | 10 | 6 | 20 | 4,1 | Agree |
| 17 Ascites | 0 | 3 | 6 | 8 | 3 | 20 | 3,55 | No |
| 18 Involvement of mesentery | 0 | 0 | 5 | 10 | 5 | 20 | 4 | Agree |
| 19 Lymphogenic metastasis | 0 | 2 | 5 | 11 | 2 | 20 | 3,65 | No |

**Supplementary document 3a.** *Total feedback of all respondents of round three (main question one)*

In your experience as a medical specialists, what factors play a role in survival and disease free survival in ovarian carcinoma **FIGO-stage IIb or higher?**

|  |  |  |  |  |  |  |  |  |  |  |  |  |  |  |
| --- | --- | --- | --- | --- | --- | --- | --- | --- | --- | --- | --- | --- | --- | --- |
|  | **Total results of round 2 (interim results)**  **n=20** | | | | | | | **Total results of round 3 (final results)**  **n=20** | | | | | | |
|  | Disagree | | Neutral | Agree | | Median | Consensus | Disagree | | Neutral | Agree | | Median | Consensus |
| Nr. Statement | 1 | 2 | 3 | 4 | 5 |  |  | 1 | 2 | 3 | 4 | 5 |  |  |
| 1 Age of the patient | 0 | 1 | 6 | 10 | 3 | 3,75 | No | 0 | 1 | 2 | 16 | 1 | 4 | Agree |
| 2 Condition of the patient | 0 | 0 | 1 | 6 | 13 | 4,6 | Agree | Consensus reached in round 2 | | | | |  | Agree |
| 3 Motivation of the patient | 0 | 3 | 4 | 7 | 6 | 3,8 | No | 0 | 1 | 7 | 8 | 4 | 4 | Agree |
| 4 Nutritional status | 0 | 0 | 0 | 16 | 4 | 4,2 | Agree | Consensus reached in round 2 | | | | |  | Agree |
| 5 Comorbidities | 0 | 0 | 0 | 13 | 7 | 4,35 | Agree | Consensus reached in round 2 | | | | |  | Agree |
| 6 WHO-performance status | 0 | 0 | 0 | 11 | 9 | 4,45 | Agree | Consensus reached in round 2 | | | | |  | Agree |
| 7 Genetic predisposition | 0 | 2 | 4 | 12 | 2 | 3,7 | No | 0 | 1 | 2 | 14 | 3 | 4 | Agree |
| 8 FIGO-classification | 0 | 0 | 1 | 9 | 10 | 3,45 | No | 0 | 0 | 0 | 8 | 12 | 5 | Agree |
| 9 Histological subtype of the tumor | 0 | 0 | 2 | 10 | 8 | 4,3 | Agree | Consensus reached in round 2 | | | | |  | Agree |
| 10 Tumor biology | 0 | 0 | 2 | 12 | 6 | 4,2 | Agree | Consensus reached in round 2 | | | | |  | Agree |
| 11 Time between chemotherapy and surgery | 1 | 3 | 4 | 8 | 4 | 3,55 | No | 1 | 2 | 4 | 12 | 1 | 4 | Agree |
| 12 Response on chemotherapy | 0 | 0 | 0 | 9 | 11 | 4,55 | Agree | Consensus reached in round 2 | | | | |  | Agree |
| 13 Dosage chemotherapy complete | 0 | 4 | 4 | 11 | 1 | 3,45 | No | 0 | 3 | 5 | 10 | 2 | 4 | Agree |
| 14 Level of PCI | 0 | 2 | 2 | 8 | 8 | 4,1 | Agree | Consensus reached in round 2 | | | | |  | Agree |
| 15 CA-125 decrease after neoadjuvant chemotherapy | 0 | 1 | 1 | 13 | 5 | 4,1 | Agree | Consensus reached in round 2 | | | | |  | Agree |
| 16 CA-125 at diagnosis | 1 | 5 | 10 | 1 | 3 | 3 | No | 1 | 5 | 7 | 6 | 1 | 3 | No |
| 17 Ascites | 0 | 4 | 6 | 6 | 4 | 3,5 | No | 0 | 2 | 6 | 9 | 3 | 4 | Agree |
| 18 Involvement of mesentery | 0 | 2 | 6 | 3 | 9 | 3,95 | No | 0 | 0 | 2 | 11 | 7 | 4 | Agree |
| 19 Pattern of metastases (lymph nodes involved) | 0 | 1 | 2 | 13 | 4 | 4 | Agree | Consensus reached in round 2 | | | | |  | Agree |
| 20 Time to recurrence | 0 | 0 | 0 | 10 | 10 | 4,5 | Agree | Consensus reached in round 2 | | | | |  | Agree |
| 21 Quality of surgical team | 0 | 0 | 6 | 11 | 3 | 3,85 | No | 0 | 0 | 1 | 15 | 4 | 4 | Agree |

**Supplementary document 3b.** *Total feedback of all respondents of round three (main question two)*

In your experience as a medical specialists, what factors play a role in survival and disease free survival in ovarian carcinoma **regardless of the FIGO-stage?**

|  | **Total results of round 2 (interim results)**  **n=20** | | | | | | | **Total results of round 3 (final results)**  **n=20** | | | | | | |
| --- | --- | --- | --- | --- | --- | --- | --- | --- | --- | --- | --- | --- | --- | --- |
|  | Disagree | | Neutral | Agree | | Median | Consensus | Disagree | | Neutral | Agree | | Median | Consensus |
| Nr. Statement | 1 | 2 | 3 | 4 | 5 |  |  | 1 | 2 | 3 | 4 | 5 |  |  |
| 1 Age of the patient | 0 | 1 | 3 | 10 | 6 | 4,05 | Agree | Consensus reached in round 2 | | | | | | Agree |
| 2 Condition of the patient | 0 | 0 | 1 | 12 | 7 | 4,3 | Agree | Consensus reached in round 2 | | | | | | Agree |
| 3 WHO-performance | 0 | 0 | 1 | 13 | 6 | 4,25 | Agree | Consensus reached in round 2 | | | | | | Agree |
| 4 Comorbidities | 0 | 0 | 2 | 13 | 5 | 4,15 | Agree | Consensus reached in round 2 | | | | | | Agree |
| 5 Smoking yes or no | 0 | 4 | 7 | 8 | 1 | 3,3 | No | 0 | 2 | 7 | 11 | 0 | 4 | Agree |
| 6 FIGO-classification | 0 | 0 | 0 | 9 | 11 | 4,55 | Agree | Consensus reached in round 2 | | | | | | Agree |
| 7 Histological subtype of the tumor | 0 | 0 | 1 | 11 | 8 | 4,35 | Agree | Consensus reached in round 2 | | | | | | Agree |
| 8 Tumor biology | 0 | 0 | 2 | 12 | 5 | 4,2 | Agree | Consensus reached in round 2 | | | | | | Agree |
| 9 Genetic predisposition | 0 | 2 | 4 | 10 | 4 | 3,8 | No | 0 | 0 | 3 | 14 | 3 | 4 | Agree |
| 10 Primary debulking achievable | 0 | 0 | 6 | 7 | 7 | 4,05 | Agree | Consensus reached in round 2 | | | | | | Agree |
| 11 Response on chemotherapy | 0 | 0 | 1 | 10 | 9 | 4,4 | Agree | Consensus reached in round 2 | | | | | | Agree |
| 12 HIPEC | 0 | 1 | 10 | 7 | 2 | 3,5 | No | 0 | 0 | 3 | 14 | 3 | 4 | Agree |
| 13 Level of CA-125 at diagnosis | 1 | 3 | 7 | 7 | 2 | 3,3 | No | 0 | 4 | 7 | 8 | 1 | 3 | No |
| 14 CA-125 decrease after neoadjuvant chemotherapy | 0 | 0 | 2 | 15 | 3 | 4,05 | Agree | Consensus reached in round 2 | | | | | | Agree |
| 15 Quality of surgical team | 0 | 0 | 5 | 12 | 3 | 3,9 | No | 0 | 0 | 1 | 15 | 4 | 4 | Agree |
| 16 Complete staging | 0 | 0 | 4 | 10 | 6 | 4,1 | Agree | Consensus reached in round 2 | | | | | | Agree |
| 17 Ascites | 0 | 3 | 6 | 8 | 3 | 3,55 | No | 0 | 0 | 7 | 11 | 2 | 4 | Agree |
| 18 Involvement of mesentery | 0 | 0 | 5 | 10 | 5 | 4 | Agree | Consensus reached in round 2 | | | | | | Agree |
| 19 Lymphogenic metastasis | 0 | 2 | 5 | 11 | 2 | 3,65 | No | 0 | 2 | 0 | 14 | 4 | 4 | Agree |

**Supplementary document 4**. *Consensus statements*

Main question 1:

In your experience as a medical specialists, what factors play a role in survival and disease free survival in ovarian carcinoma **FIGO-stage IIb or higher?**

| Agreement (median score 4 or 5 on the Likert Scale) | Consensus reached in round: |
| --- | --- |
| Age of the patient  Condition of the patient  Motivation of the patient  Nutritional status  Comorbidities  WHO-performance status  Genetic predisposition  FIGO-classification  Histological subtype of the tumor  Tumor biology  Time between chemotherapy and surgery  Response on chemotherapy  Dosage chemotherapy complete  Level of PCI  CA-125 decrease after neoadjuvant chemotherapy  Ascites  Involvement of mesentery  Pattern of metastases (lymfenodes involved)  Time to recurrence  Quality of surgical team | 3  2  3  2  2  2  3  3  2  2  3  2  3  2  2  3  3  2  2  3 |

No consensus reached for the following statement(s):

Level of CA-125 at diagnosis

**Supplementary document 4b**. *Consensus statements*

Main question 2:

In your experience as a medical specialists, what factors play a role in survival and disease free survival in ovarian carcinoma **regardless of the FIGO-stage?**

| Agreement (median score 4 or 5 on the Likert Scale) | Consensus reached in round: |
| --- | --- |
| Age of the patient  Condition of the patient  WHO-performance  Comorbidities  Smoking yes or no  FIGO-classification  Histological subtype of the tumor  Tumor biology  Genetic predisposition  Primary debulking achievable  Response on chemotherapy  HIPEC  CA-125 decrease after neoadjuvant chemotherapy  Quality of surgical team  Complete staging  Ascites  Involvement of mesentery  Lymphogenic metastasis | 2  2  2  2  3  2  2  2  3  2  2  3  2  3  2  3  2  3 |

No consensus reached for the following statement(s):

Level of CA-125 at diagnosis
