# Supplementary material for: Ovarian cancer recurrence prediction: comparing confirmatory to real-world predictors with machine learning
Source: ESMO Real World Data Digit Oncol. 2026 Jan 8;11:100666. doi: 10.1016/j.esmorw.2025.100666 (PMC13040900; doi:10.1016/j.esmorw.2025.100666)
Supplement: Supplementary Appendix [file mmc2.docx]

**Appendix**

1. Durations of different therapy regimes

Different chemotherapy regimens showed different total therapy durations which indicates an immortal time bias (cf. Figure A1).


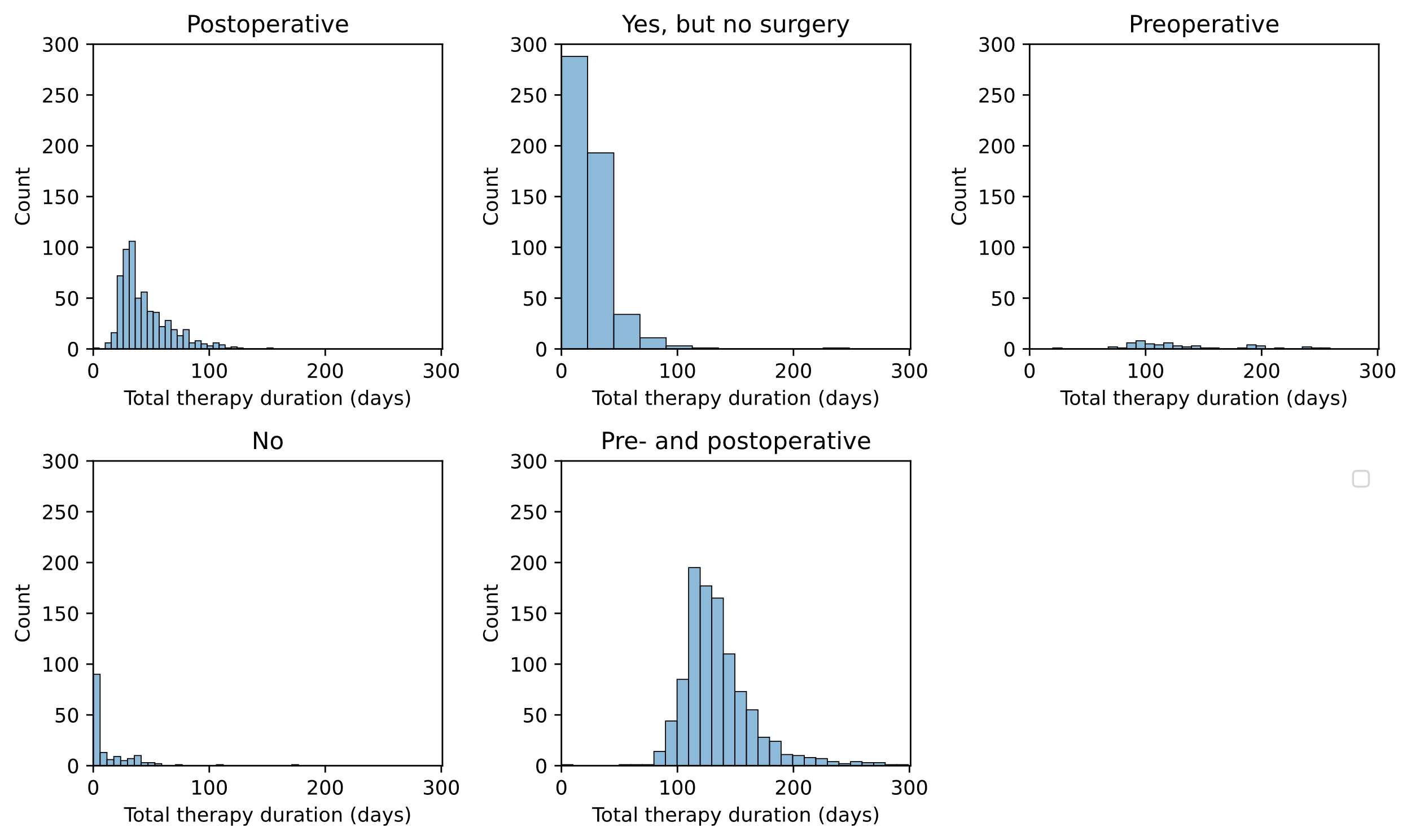


*Figure A1: Histograms of durations of different chemotherapy regimens before the exclusion of 355 patients who had recurrence up to 5 months since diagnosis. Each regime is given in the title of each plot.*

1. Model fitting and variable selection

All Cox-based models were fitted through the “scikit-survival” python package (v. 0.23.0).

- 1. Missing value imputation

We used a Missforest custom algorithm for missing value imputation [25],which is a popular non-parametric ML method based on random forests. The variables with more than 30% missing values were not imputed. Imputation was done by using all the predictor set, separately for the Delphi and the data-driven variable approach.

- 1. Adjustment for deterministic zero values

To address the issue of zero-inflation, we introduced two dummy variables: one to account for cases where no adjuvant chemotherapy was administered, and another where no lymph nodes were examined. These dummy variables were used to separate zeros that arise from specific clinical situations (such as the absence of chemotherapy or lymph node examination) from zeros that hold statistical significance, ensuring more accurate modeling. These variables did not contribute to the interpretation of the primary findings.

- 1. Data processing

Preprocessing took place in “scikit-learn” python package (v. 1.4.2). Ordinal and categorical variables were transformed with one-hot encoding. Numerical variables were rescaled to be within [0,1] with the minmaxscaler.

- 1. Parameter optimization

We used randomized parameter search based on 10 cross-validation folds to find the best model parameters. For the Lasso model, the alpha regularization parameter was optimized using a range estimated from the data, following to the default procedure of the “scikit-survival” python package, which evaluates a 100 different alpha values with increments of 0.01. For the Elastic net model, the alpha parameter was estimated similarly to the Lasso model. The parameter determining the balance between L1 and L2 regularization, was optimized using a range between 0.1 and 0.9, with increments of 0.1.

- 1. Internal validation procedure

We fitted the ML models following the internal validation procedure laid out in [26].. According to internal validation, the generalization error (i.e., overfitting) is estimated from the original data using bootstrapping. This procedure has the advantage over traditional train-test splits of the data, as it preserves predictive power. Specifically, we randomly sampled patients from the original data set with replacement and we created 500 independent data sets. For each of these, we repeated the modeling steps independently (i.e., missing value imputation, model fitting and parameter optimization). We measured performance of each independently-fitted model on the bootstrapped data and the original data, the difference of which constitutes the generalization error (i.e., 500 generalization error rates). Subsequently, by subtracting the lower and upper quantiles of the generalization error data that contain 95% of the distribution from the model performance on the whole original data set, we estimated the lower and upper bound of the generalization-error-adjusted performance.

- 1. Variable selection

For variable selection, we used the best parameters from model fitting after each bootstrapping iteration based on the bootstrapped data. This has two advantages: first, by using the bootstrapped data we perform variable selection for the underlying population and not just the specific cohort data set, and second, it allows us to quantify our uncertainty regarding variable selection and perform inference. Specifically, we quantified how many times each coefficient was set to zero through the 500 bootstrap iteration, yielding a mean variable selection percentage and a corresponding confidence interval around it.

- 1. RSFs and XGBoost models

Both RSFs and XGBoost are ensemble tree methods. RSFs use a collection of weak classifiers (i.e., decision trees) whose combined prediction is superior than each single one. Unlike RSFs, it iteratively grows decision trees and optimizes on the residual error after each iteration. We followed the same model building steps as we did in the case of Cox models previously, however with the exception that we fitted the XGBoost model to data imputed by Missforest and to data with missing values (thereby performing no imputation). XGBoost can natively handle missing values, making imputation prior to model fitting not necessary. This way, therefore, we tested whether imputation impacted the performance of the XGBoost. The RSFs were fitted through the “scikit-survival” package and the XGBoost through the python package “py-xgboost” (v. 2.0.3).

The parameter range of XGBoost on which we performed randomized cross-validation seach was the following: number of estimators within [10, 20, 50, 100, 200], max tree depth within [2, 3, 4, 5, 6, 7, 8], minimum samples for branch split within integer range of [5, 6, 7, …, 50] and minimum samples for final leaf within integer range of [1,2,3, …, 25].

The parameter range of RSFs was similar as for the XGBoost: number of estimators within [10, 20, 50, 100, 200] , max tree depth within [2, 3, 4, 5, 6, 7, 8], minimum samples for branch split within integer range of [5, 6, 7, …, 50] and minimum samples for final leaf within integer range of [1,2,3, …, 25].

1. Calibration of the Elastic Net model

While c-index indicates how well a model discriminates the recurrence risk of patients against other models, calibration is also important to be evaluated because it captures how well the data are fitted by the model (cf. [26]). Model calibration of the Elastic Net model is shown in Figure D1. The calibration is derived from the bootstrap samples, specifically it shows well the survival function of the original data set is approximated from the models fitted on the bootstrap samples. Evidently, no gross misfit was observed.


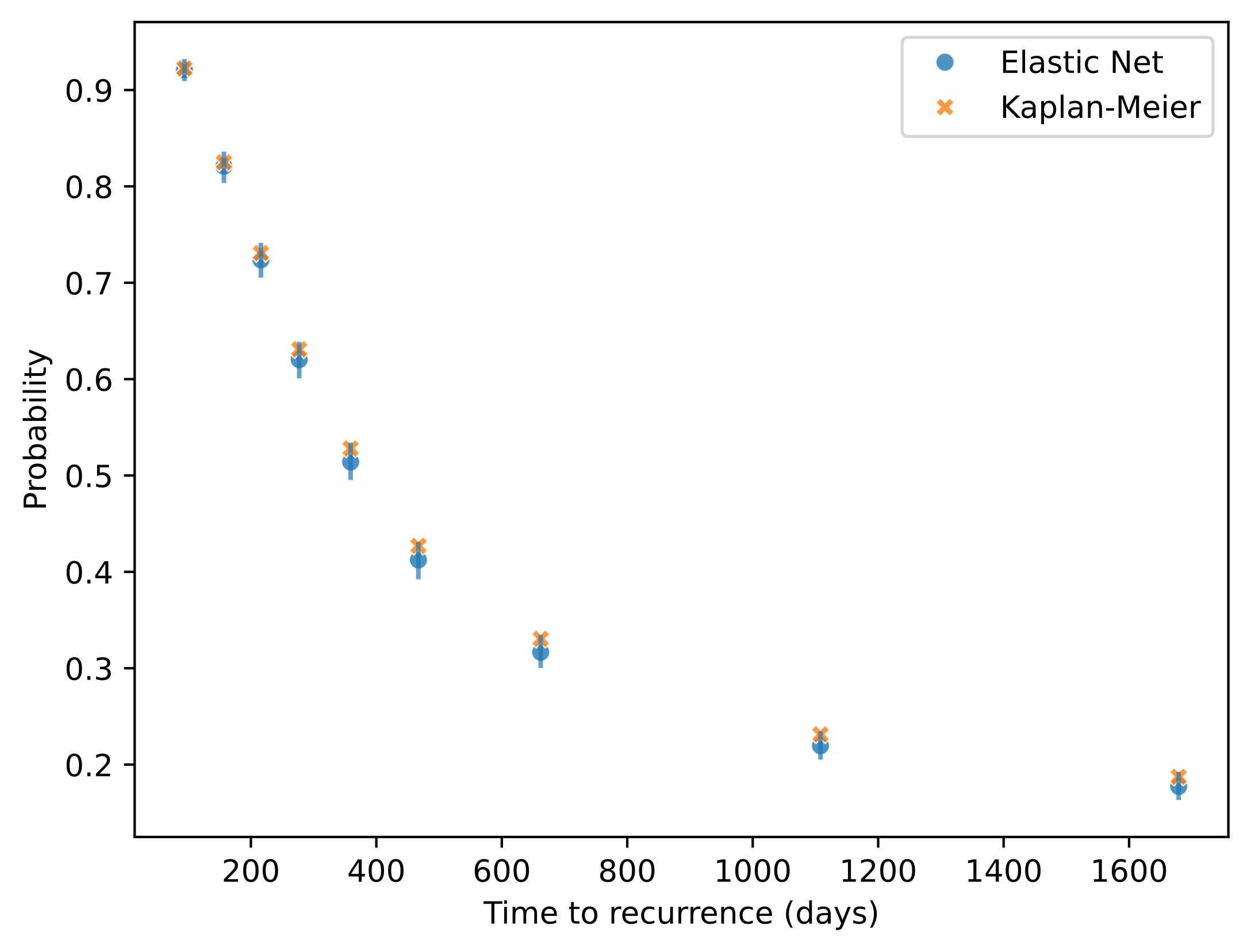


*Figure D1. Calibration of the Elastic Net model. Kaplan-Meier shows progression-free survival probabilities for 9 deciles (10% - 90%) of original data set. In addition, respective Elastic Net estimates are also shown based on bootstrap samples (average and CI_95%_ quantile-based intervals included).*
